# Coronavirus Exploitation of Ubiquitination and Selective Autophagy to Control Innate Immunity: Mechanisms, Systems Logic, and Therapeutic Opportunities

**DOI:** 10.3390/v18101068

**Published:** 2026-09-26

**Authors:** Pan Wang, Yuxi Zhao, Yuqiu Yang, Juan Zhang, Junfang Wu, Jiandong Wang

**Affiliations:** 1 Institute of Animal Science, Ningxia Academy of Agricultural and Forestry Sciences, Yinchuan 750002, China; wp_1112@163.com (P.W.); zyxwpc18214@gmail.com (Y.Z.); 2 School of Animal Science and Technology, Ningxia University, Yinchuan 750021, China; yangyuqiu0729@163.com (Y.Y.); zja1623@163.com (J.Z.); wujunfang0813@163.com (J.W.)

**Keywords:** coronavirus, SARS-CoV-2, ubiquitin, deubiquitination, ISGylation, selective autophagy, virophagy, mitophagy, innate immunity, interferon

## Abstract

*Coronaviruses* replicate in membrane-rich cytoplasmic niches while confronting innate immune systems whose activation, signal propagation, and termination are extensively controlled by ubiquitin and autophagy. These two regulatory systems are often discussed separately. Yet they form a coupled information-and-disposal network: ubiquitin chains encode the identity and fate of immune proteins or viral cargo, whereas selective autophagy receptors read these signals and route substrates to lysosomes. *Coronaviruses* exploit this coupling at several levels. Papain-like proteases remove ubiquitin and *ISG15* from antiviral factors; structural, nonstructural, and accessory proteins redirect host ubiquitin ligases or destabilize immune adaptors; and viral proteins remodel autophagosome biogenesis, mitophagy, lysosome function, and selective virophagy. These activities do more than globally suppress immunity. They delay early interferon production, reshape inflammatory signaling, preserve replication organelles, adjust mitochondrial quality control, and protect viral proteins from cargo-selective degradation. Importantly, the outcome is context dependent. Autophagy initiation can support membrane supply or suppress mitochondrial antiviral signaling, whereas completion of autophagic flux can eliminate viral material and restrain damaging inflammation. Likewise, K48-linked ubiquitination may remove antiviral proteins, but nondegradative K63- or M1-linked chains can either assemble antiviral signalosomes or promote pathological NF-κB activity. Here, we synthesize evidence from human and animal *coronaviruses* to propose that viral immune evasion is governed by spatially and temporally selective rewiring rather than indiscriminate inhibition. We distinguish mechanisms demonstrated during infection from those inferred from ectopic expression, identify points of convergence among RIG-I-like receptor, cGAS-STING, NF-κB, inflammasome, and autophagy pathways, and discuss why apparently contradictory observations often reflect differences in flux, cell type, infection stage, and viral lineage. This integrated view highlights viral proteases, host E3 ligase-substrate interfaces, autophagy receptors, and organelle contact sites as therapeutic nodes, while emphasizing that interventions must preserve the homeostatic functions of ubiquitin and autophagy.

## 1. Introduction

The outcome of coronavirus infection is decided before adaptive immunity reaches full strength. Airway and intestinal epithelial cells, tissue-resident macrophages, dendritic cells, and recruited myeloid populations detect viral RNA, membrane damage, mitochondrial stress, and cell injury. These inputs converge on type I and III interferons, nuclear factor-κB (NF-κB), stress pathways, inflammasomes, and cell-death programs. A rapid, spatially restricted response can limit replication with tolerable tissue damage. A delayed response, followed by broad inflammatory activation, permits viral expansion while increasing immunopathology. This temporal mismatch was a defining feature of severe acute respiratory syndrome coronavirus 2 (SARS-CoV-2) infection and is also evident, in different forms, in infections caused by SARS-CoV, Middle East respiratory syndrome *coronavirus* (MERS-CoV), and pathogenic livestock *coronaviruses* [1,2,3,4].

Ubiquitination and autophagy occupy a central position in this contest. Ubiquitin is not merely a proteasomal destruction tag. E1 activating enzymes, E2 conjugating enzymes, E3 ligases, deubiquitinating enzymes (DUBs), and ubiquitin-binding proteins build and interpret chains linked through Lys6, Lys11, Lys27, Lys29, Lys33, Lys48, Lys63, or the amino-terminal methionine. Chain topology, length, branching, and substrate location determine whether ubiquitination promotes degradation, membrane trafficking, protein complex assembly, kinase activation, DNA repair, or organelle quality control [5,6]. Antiviral pathways are particularly dependent on nondegradative ubiquitin scaffolds. K63-linked chains help activate retinoic acid-inducible gene I (RIG-I), mitochondrial antiviral-signaling protein (MAVS), TANK-binding kinase 1 (TBK1), and inhibitor of NF-κB kinase complexes; M1-linked chains stabilize NF-κB signalosomes; and K27- or K63-linked modifications contribute to stimulator of interferon genes (STING) activation. K48-linked or mixed chains, by contrast, frequently terminate signaling by proteasomal removal. The same protein can therefore be activated or silenced by different chain architectures.

Macroautophagy, hereafter autophagy, encloses cytoplasmic material in double-membrane autophagosomes and delivers it to lysosomes. Selective autophagy adds molecular discrimination to this bulk pathway. Cargo receptors such as SQSTM1/p62, NBR1, CALCOCO2/NDP52, OPTN, TAX1BP1, and CCDC50 bind ubiquitinated cargo and ATG8-family proteins through ubiquitin-binding regions and LC3-interacting regions. Receptor phosphorylation, cargo condensation, membrane contact, and engagement of the FIP200-ULK1 initiation machinery determine whether a damaged organelle, protein aggregate, bacterium, or virion is captured [7,8,9,10]. Thus, ubiquitin writes a molecular address and selective autophagy reads it. This coupling makes the pathway both an antiviral effector, often termed virophagy when viral material is targeted, and an immune regulator that removes signaling platforms or damaged mitochondria.

*Coronaviruses* are unusually well positioned to manipulate this network. Their large positive-sense RNA genomes encode two polyproteins that are cleaved into nonstructural proteins (nsps), four principal structural proteins, and lineage-specific accessory proteins. Replication occurs on endoplasmic reticulum (ER)-derived double-membrane vesicles (DMVs), placing viral biogenesis close to ER quality-control machinery and membrane sources used by autophagy [11,12]. The nsp3 papain-like protease (PLpro) is simultaneously a viral polyprotein-processing enzyme and a DUB/deISGylase. Other viral products contact E3 ligases, autophagy regulators, mitochondrial adaptors, and endolysosomal trafficking proteins. Proteomic maps have shown that coronavirus proteins engage an extensive host network enriched for ubiquitin, vesicle transport, mitochondria, lipid metabolism, and protein-quality-control factors [13,14,15].

The literature nevertheless contains an apparent paradox. Autophagy inhibition sometimes reduces coronavirus replication, suggesting that the virus uses the pathway. In other systems, enhancing autophagic flux limits replication, suggesting antiviral clearance. Viral proteins can induce LC3 lipidation while simultaneously blocking autophagosome-lysosome fusion. Mitophagy can protect cells from inflammatory mitochondrial damage, yet it can also remove MAVS-bearing mitochondria and suppress interferon. These are not necessarily contradictory findings. “Autophagy” encompasses initiation, membrane expansion, cargo selection, fusion, lysosomal degradation, and recycling. A virus may promote one module and inhibit another. The immunological consequence also changes across time: reducing MAVS signaling early benefits the virus, whereas removing damaged mitochondria later may protect the host from inflammasome-driven injury.

This review is a narrative synthesis of the literature. Relevant articles were identified through PubMed, supplemented by GeenMedical and Baidu Scholar, using keywords such as “coronavirus”, “SARS-CoV-2”, “ubiquitin”, “autophagy”, “interferon”, and related terms. The search covered literature available up to the time of manuscript preparation, without a strict time limit. Peer-reviewed English-language articles relevant to coronavirus–host interactions were included; articles without full text, conference abstracts, non-English publications, and duplicates were excluded. Reference lists of key articles were also screened. As a narrative review, no formal systematic search protocol was used.

Although MERS-CoV possesses unique accessory proteins and distinct immune evasion strategies, the specific mechanisms by which it manipulates the ubiquitin-autophagy network remain relatively underexplored compared to SARS-CoV-2 and related animal *coronaviruses*. Therefore, this review primarily focuses on SARS-CoV-2 and animal *coronaviruses* to provide a comprehensive mechanistic synthesis.

To make this comparative scope explicit, we include representative viruses from different coronavirus genera: PEDV (*Alphacoronavirus*), IBV (*Gammacoronavirus*), PDCoV (*Deltacoronavirus*), and SARS-CoV, SARS-CoV-2, and MERS-CoV (*Betacoronavirus*). Findings obtained only in animal *coronaviruses* are identified as such and are not directly extrapolated to SARS-CoV-2. This cross-genus comparison highlights both conserved principles and lineage-specific differences in ubiquitin–autophagy manipulation.

This Review advances three arguments. First, coronavirus manipulation of ubiquitin and selective autophagy is best understood as a connected regulatory system rather than two parallel topics. Second, viral success depends on selective rewiring in space and time: productive membrane and quality-control functions are retained, while antiviral cargo recognition and immune signal propagation are weakened. Third, therapeutic intervention requires mechanistic precision. Global proteasome or autophagy modulation is likely to have opposing effects in infected and bystander cells; viral catalytic activities and virus-induced host interfaces offer more selective targets.

## 2. Ubiquitin and Selective Autophagy as an Integrated Innate Immune Circuit

Innate signaling is organized around transient, membrane-associated supramolecular complexes. During RNA virus infection, RIG-I and melanoma differentiation-associated protein 5 (MDA5) detect distinct viral RNA features. RIG-I activation depends on conformational exposure and K63-linked ubiquitin, produced primarily by TRIM25 and RIPLET/RNF135 in a context-dependent manner [16,17,18]. Activated receptors nucleate MAVS filaments on mitochondria and mitochondria-associated membranes. MAVS recruits TRAF proteins, TBK1, IKKepsilon, and the canonical IKK complex, resulting in IRF3/IRF7 and NF-κB activation. Ubiquitin chains are integral structural components of this signalosome, not passive decorations. Similar logic governs Toll-like receptor and interleukin-1 receptor pathways, in which TRAF6, cIAPs, LUBAC, and ubiquitin-binding adaptors assemble kinase platforms.

Although *coronaviruses* are RNA viruses, DNA-sensing pathways also contribute. Mitochondrial stress and cell damage can release mitochondrial DNA, and membrane fusion or nuclear injury may generate additional cGAS ligands. cGAS produces cyclic GMP-AMP, which activates ER-resident STING. STING traffics from the ER to ER-Golgi intermediate compartments and the Golgi. This trafficking permits TBK1 recruitment and IRF3 activation. Ubiquitination regulates almost every step, including STING stability, oligomerization, trafficking, and termination [19]. This creates an indirect route by which coronavirus-driven organelle damage becomes an antiviral signal.

Autophagy intersects these pathways in at least four ways. First, it can directly remove viral components. Ubiquitinated capsids, nucleoproteins, or replication-associated material are recognized by selective receptors and delivered to lysosomes. Second, it controls the abundance and duration of immune signaling complexes. p62, NDP52, TAX1BP1, and OPTN can target ubiquitinated signaling proteins for degradation. Conversely, autophagy deficiency causes p62 accumulation. This may amplify or distort NF-kB signaling. Third, mitophagy removes damaged mitochondria. This limits mitochondrial reactive oxygen species (mtROS), mitochondrial DNA release, NLRP3 inflammasome activation, and cell death. Fourth, autophagy proteins have noncanonical signaling roles. ATG5-ATG12, ATG16L1, LC3 conjugation machinery, and autophagy initiation complexes can regulate interferon and inflammatory pathways independently of complete lysosomal flux [20,21].

Selective autophagy itself relies on ubiquitin editing. Damaged mitochondria provide the clearest model. Loss of membrane potential stabilizes PINK1 on the outer mitochondrial membrane, enabling phosphorylation of ubiquitin and activation of the E3 ligase Parkin. Amplified phospho-ubiquitin chains recruit OPTN and NDP52, which in turn recruit TBK1 and the autophagy machinery [22,23,24]. Receptor-mediated mitophagy can also proceed through BNIP3, BNIP3L/NIX, or FUNDC1 without a canonical ubiquitin coat, particularly during hypoxia or metabolic remodeling. These pathways are not interchangeable. PINK1-Parkin mitophagy strongly couples damage sensing to ubiquitin, whereas receptor-mediated mitophagy can be driven by transcriptional or phosphorylation-dependent changes.

The topology of ubiquitin chains therefore determines both signaling and disposal. K63 chains on RIG-I promote activation, but K63 chains on damaged mitochondria promote autophagic capture. K48 chains can eliminate a signaling adaptor through the proteasome, whereas aggregated or oligomeric K48-ubiquitinated proteins may also be handled by p62-dependent autophagy. M1 chains recruit NEMO and stabilize NF-κB signaling, but prolonged signaling complexes may later be removed through TAX1BP1-dependent autophagy. Viral DUBs can erase these marks, viral proteins can recruit host E3 ligases to install alternative chains, and viral disruption of lysosomal flux can prevent the final disposal step. The relevant question is consequently not whether ubiquitination or autophagy increases during infection. Instead, it is which substrate carries which chain, where it is located, which receptor reads it, and whether flux reaches a functional lysosome.

This systems view explains why static measurements are inadequate. Increased LC3-II may reflect increased autophagosome production or blocked degradation. Reduced abundance of an immune protein may result from proteasomal degradation, lysosomal degradation, translational arrest, or viral protease cleavage. Co-localization of a viral protein with LC3 does not establish selective autophagic capture. Likewise, an interaction found by affinity purification does not demonstrate that it occurs at endogenous abundance or during productive infection. Strong mechanistic evidence requires several approaches. These include chain-specific ubiquitin analysis, lysosomal and proteasomal rescue, autophagic flux measurements, receptor genetics, viral mutants, infection-competent systems, and ideally in vivo validation. A summary of the functional logic of different ubiquitin signals in coronavirus infection is provided in Table 1.

## 3. Viral Editing of Ubiquitin-Dependent Antiviral Signaling

### 3.1. PLpro: A Catalytic Bridge Between Replication and Immune Evasion

PLpro is the most direct coronavirus-encoded regulator of ubiquitin. Embedded within nsp3, it cleaves the viral polyprotein to release nsp1, nsp2, and nsp3. The same catalytic center recognizes ubiquitin-like folds and removes ubiquitin or interferon-stimulated gene 15 (*ISG15*) from host proteins. Structural studies of SARS-CoV and SARS-CoV-2 PLpro revealed a papain-like catalytic architecture with substrate-binding surfaces that discriminate among viral cleavage sequences, ubiquitin, and *ISG15* [26,27,29,30]. SARS-CoV-2 PLpro shows a pronounced preference for *ISG15* relative to many ubiquitin substrates, whereas related coronavirus enzymes differ in linkage selectivity and substrate bias. These differences matter because DUB and deISGylase activity can uncouple viral polyprotein processing from host immune antagonism.

*ISG15* is induced by interferon and conjugated to proteins by an E1-E2-E3 cascade involving UBA7, UBE2L6, and HERC5. ISGylation can alter protein stability, localization, or activity and restrict multiple viruses. SARS-CoV-2 PLpro deISGylates host proteins and processes pro-*ISG15*, thereby weakening cell-intrinsic antiviral effects while potentially increasing extracellular free *ISG15*, which can stimulate cytokine production by immune cells. Shin and colleagues showed that PLpro preferentially cleaves *ISG15* and attenuates type I interferon responses; pharmacological inhibition restored antiviral signaling and reduced viral replication [26]. This dual action suggests a mechanism for the combination of weak early interferon and excessive later inflammation: intracellular deISGylation benefits replication, whereas altered extracellular *ISG15* may contribute to immune activation. The balance is species dependent because PLpro recognizes human and mouse *ISG15* differently, complicating interpretation of murine models.

PLpro also antagonizes ubiquitin-dependent signaling at several nodes. Coronavirus PLpro proteins have been reported to reduce ubiquitination or activation of RIG-I, MDA5-associated pathways, MAVS, TRAF3, TRAF6, TBK1, IRF3, and STING, although substrate assignments and enzyme preferences differ among viral species and experimental systems [28,31,32,33]. The broad conclusion—that PLpro suppresses interferon and NF-κB signaling—is robust. The exact endogenous substrate hierarchy is less settled. Overexpressed PLpro can access proteins that may not encounter nsp3 at replication organelles during infection. In addition, catalytic mutants may alter nsp3 conformation or polyprotein processing. Infection with separation-of-function viral mutants is therefore essential for distinguishing direct deconjugation from secondary consequences.

PLpro connects ubiquitin editing to autophagy. Coronavirus PLpro cleaves ULK1, the kinase that initiates canonical autophagy, disrupting formation of a functional autophagy initiation complex [34]. Cleavage of ULK1 can limit antiviral autophagic degradation while affecting innate signaling functions of ULK1. PLpro-mediated deubiquitination may also alter the ubiquitin coats required for virophagy or mitophagy, although direct substrate-level evidence during SARS-CoV-2 infection remains incomplete. Conceptually, this is an economical viral strategy: the same protease that matures the replication machinery removes immune-activating conjugates and disables a key gateway to lysosomal clearance.

### 3.2. Host E3 Ligases Are Redirected Rather than Simply Inhibited

Coronavirus proteins also exploit host ubiquitin ligases. Affinity-proteomic studies identified interactions between SARS-CoV-2 proteins and Cullin-RING ligases, HECT ligases, TRIM proteins, and ubiquitin adaptors [13,14,15]. Some interactions control viral protein abundance. For example, a Cullin 5-based complex regulates ORF9b stability and thereby influences SARS-CoV-2 replication [35]. This illustrates a recurring principle: host ubiquitination is not uniformly antiviral. Degradation of a viral antagonist can protect the host, whereas controlled turnover can optimize viral protein stoichiometry, prevent toxic accumulation, or create temporally restricted immune suppression.

Viral proteins can also redirect ligases toward host targets. SARS-CoV-2 ORF3a has been reported to promote STUB1/CHIP-mediated ubiquitination and proteasomal degradation of PTEN, weakening antiviral interferon signaling [36]. Other coronavirus proteins recruit or influence E3 ligases that target MAVS, TBK1, IRF3, STAT proteins, or regulators of NF-κB. In several cases, however, the evidence comes from transfection systems, and the physiological importance of the proposed ligase-substrate pair awaits recombinant-virus genetics. The mechanistic standard should include loss of the ligase, identification of modified lysines and chain types, rescue by ubiquitination-resistant substrate mutants, and demonstration that the interaction changes viral fitness in relevant primary cells.

The nucleocapsid (N) protein illustrates how ubiquitin-dependent recognition can be blocked without enzymatic deubiquitination. SARS-CoV N protein interferes with TRIM25-mediated activation of RIG-I, and SARS-CoV-2 N has been reported to bind RIG-I pathway components, alter stress granules and condensates, and suppress interferon production [37,38]. Because N is an abundant RNA-binding protein that undergoes phosphorylation and liquid–liquid phase separation, it may sequester RNA or signaling proteins away from productive ubiquitin-driven complexes. This mechanism is biophysically distinct from PLpro catalysis: rather than erase a chain, N changes the local concentration, accessibility, and material state in which ubiquitin-dependent activation occurs.

Ubiquitin remodeling can also be proinflammatory. Coronavirus nsp14 induces NF-κB activity through linear ubiquitination, engaging LUBAC-dependent signaling [25]. This observation challenges a simplistic model of global immune suppression. A virus benefits most from selectively reducing antiviral interferon while preserving or redirecting inflammatory programs that promote tissue damage, immune-cell recruitment, or transmission. The separation is possible because RIG-I-MAVS-IRF3 and NF-κB share adaptors but depend on distinct subcellular assemblies and ubiquitin codes. Timing is also decisive: weak interferon early and exaggerated NF-κB or inflammasome activity later can coexist in the same infection.

### 3.3. Mitochondrial Ubiquitin Signaling as a Strategic Target

Mitochondria are both metabolic organelles and antiviral signaling platforms. MAVS resides on the outer membrane, while TOM70 supports antiviral signaling by linking mitochondrial protein import machinery to HSP90-bound TBK1 and IRF3. SARS-CoV-2 ORF9b localizes to mitochondria and binds TOM70, suppressing interferon induction [39,40]. Structural work revealed the ORF9b-TOM70 interface and suggested that viral occupation changes TOM70 conformational or chaperone interactions. ORF9b also affects mitochondrial morphology and quality control. Its abundance is itself regulated by ubiquitination, establishing a feedback loop in which host ligases tune a viral antagonist that operates on a ubiquitin- and autophagy-rich organelle.

SARS-CoV-2 nsp8 can damage mitochondria and induce mitophagy [41]. Productive infection likewise changes mitochondrial morphology, respiration, and gene expression. If damaged mitochondria are rapidly removed, MAVS platform abundance and mtDNA-driven cGAS-STING activation decrease. The result can be reduced interferon despite improved short-term cellular viability. Conversely, incomplete mitophagy permits accumulation of mtROS and mitochondrial DNA, supporting NLRP3 activation and inflammatory cell death. Coronavirus control of mitochondrial quality is therefore a rheostat, not a binary switch. The virus must suppress antiviral signaling without destroying the bioenergetic and membrane functions required for replication too early.

Evidence from porcine deltacoronavirus (PDCoV) strengthens this model: infection triggers mitophagy, dampens interferon, and promotes replication [42]. Yet extrapolation across coronavirus genera requires caution. Viral proteins, tissue tropism, receptor usage, and baseline autophagic activity differ. Moreover, mitophagy observed in immortalized cells under high multiplicity may reflect terminal damage rather than an evolved immune-evasion program. Time-resolved genetic interruption of specific mitophagy branches is needed to distinguish adaptive viral manipulation from a host stress response. A detailed summary of representative coronavirus proteins that rewire these pathways is provided in Table 2.

## 4. Selective Autophagy as Antiviral Defense and Viral Resource

### 4.1. Virophagy and the Problem of Cargo Recognition

Selective autophagy can capture complete virions, capsids, nucleoproteins, or replication complexes. For many viruses, p62, NDP52, OPTN, TAX1BP1, or newly recognized receptors bind ubiquitinated viral cargo and recruit LC3/GABARAP proteins. This process can reduce viral burden directly and enhance antigen presentation or inflammatory signaling. Viruses counter it by removing ubiquitin, masking receptor-binding motifs, cleaving receptors, or blocking lysosomal delivery [7,8,9,10,49].

For *coronaviruses*, direct virophagy is increasingly supported by animal-virus studies. RBM10 restricts porcine epidemic diarrhea virus (PEDV) by promoting selective autophagic degradation of nsp3 [50]. PGAM5 has been reported to suppress PEDV through selective autophagy-mediated degradation of N protein [51]. ZNF219 promotes degradation of the PEDV S2 protein [52]. In PDCoV, NBR1 recognizes the envelope (E) protein and routes it toward autophagic degradation; the viral main protease nsp5 cleaves NBR1 and thereby disrupts this defense [53]. More recent work identifies CCDC50 as another receptor targeting PDCoV E, again antagonized by nsp5 cleavage [54]. These studies reveal a layered evolutionary contest: host proteins recognize distinct coronavirus components, while the viral protease attacks the receptor rather than the cargo.

The importance of these findings extends beyond veterinary virology. They show that coronavirus structural and nonstructural proteins can become selective-autophagy cargo and that viral proteases evolved receptor-specific countermeasures. However, these virophagy mechanisms are based mainly on PEDV, PDCoV, and IBV, and should not be directly extrapolated to SARS-CoV-2 without sufficient evidence. Although these specific receptors (NBR1, CCDC50, RBM10, PGAM5, and ZNF219) have been well characterized in animal *coronaviruses*, their definitive roles and functions in SARS-CoV-2-infected human cells remain largely unexplored, highlighting a critical translational gap. Therefore, they are discussed here as comparative evidence rather than established SARS-CoV-2 mechanisms. Cargo selectivity is shaped by physical state. N protein forms RNA-rich condensates, and p62 is itself a phase-separating ubiquitin receptor. Condensation could facilitate autophagic capture by concentrating ubiquitinated N, or protect N by forming material states inaccessible to receptors and membranes. Phosphorylation, ubiquitination, and RNA occupancy will determine which outcome prevails. The same reasoning applies to replication organelles: a DMV can shield viral RNA from RIG-I and autophagy, but damaged or incompletely sealed membranes may expose ubiquitinated viral or host components that trigger selective capture.

### 4.2. Coronavirus Remodeling of Autophagosome Biogenesis

*Coronaviruses* extensively remodel ER membranes to produce DMVs. Early work with infectious bronchitis virus and related *coronaviruses* implicated nsp6 in generating autophagosome-like membranes while restricting their expansion [46]. SARS-CoV-2 nsp6 likewise reduces autophagosome size, with the sigma-1 receptor contributing to this phenotype and to viral replication [47]. A small-autophagosome state may be advantageous because it supplies membrane intermediates without allowing efficient sequestration of large viral replication structures or organelles. Recent work further suggests that coronaviral nsp6 engages ER-associated degradation machinery to mobilize lipids for DMV growth [48]. This connects ubiquitin-dependent ER quality control, lipid metabolism, and replication-organelle biogenesis.

The relationship between DMVs and canonical autophagosomes is not one of simple identity. *Coronaviruses* can use LC3-related or ER-derived membrane processes without requiring the complete ATG pathway, and requirements vary by virus and cell type. Some early studies found coronavirus replication independent of ATG5, whereas others identified roles for LC3, autophagy regulators, or lipid-remodeling pathways. The most coherent interpretation is modular appropriation: the virus borrows membrane supply, lipid transfer, or curvature machinery but does not necessarily build conventional degradative autophagosomes.

SARS-CoV-2 infection alters autophagy at multiple levels. Multi-omic and functional screens show changes in autophagy regulators, lysosomal biogenesis, lipid metabolism, and phosphorylation networks [14,15,55]. Pharmacological induction of autophagic flux can reduce replication in some cell systems, while inhibitors of initiation can also impair replication in others. Drug pleiotropy contributes substantially to these differences. Chloroquine and related lysosomotropic agents affect endosomal pH, entry, lysosome function, and immune signaling, not just autophagy. mTOR, AMPK, and VPS34 inhibitors likewise influence translation, metabolism, and membrane trafficking. Genetic perturbation of defined steps, combined with viral life-cycle measurements, is therefore more informative than labeling a compound an “autophagy inhibitor.”

### 4.3. Blocking Completion: ORF3a and the Autophagosome-Lysosome Checkpoint

SARS-CoV-2 ORF3a is a potent inhibitor of autophagosome maturation. Studies have shown that ORF3a interacts with or functionally disrupts VPS39 and the HOPS tethering complex, preventing productive autophagosome-lysosome fusion [43,44]. ORF3a also perturbs Rab7 cycling and lysosomal function through TBC1D5-dependent mechanisms [45]. The consequence is accumulation of autophagosomes with impaired degradative flux. This phenotype can be misread as “autophagy induction” if only LC3-II or puncta are measured.

Blocking fusion provides several advantages. Viral material that enters autophagosomes avoids lysosomal destruction. Autophagic membranes and cargo receptors accumulate, potentially altering membrane traffic and inflammatory signaling. Damaged mitochondria may initiate sequestration but fail to be fully degraded, creating a pool of stressed organelles that can release inflammatory mediators. Lysosomal dysfunction also affects cholesterol export, antigen processing, plasma-membrane repair, and cell death. Thus, ORF3a converts an antiviral disposal pathway into a stalled compartment that may simultaneously protect viral cargo and promote pathology.

ORF3a does not act alone. PLpro cleavage of ULK1 can suppress initiation [34], nsp6 restricts autophagosome expansion [46,47], and other viral proteins influence lysosomal acidification or trafficking. At first glance, inhibiting both initiation and completion seems redundant or contradictory. Spatial and temporal specialization resolves the issue. Early in infection, limited initiation and small autophagosomes may support membrane remodeling while avoiding virophagy. Later, fusion blockade prevents any captured viral material from being destroyed. Different proteins may also act in different cell compartments or at different expression thresholds. The aggregate phenotype is not a uniform block but a rerouted flux optimized for replication.

### 4.4. Mitophagy: Immune Silencing Versus Damage Control

SARS-CoV-2 can stimulate autophagy or mitophagy to suppress type I interferon. Hui and colleagues reported that viral infection promotes autophagy-dependent degradation of MAVS, thereby reducing interferon output [56]. Nsp8-induced mitochondrial damage and mitophagy provides a complementary route [41]. The key mechanistic concept is selective removal of the signaling organelle or adaptor rather than global degradation of cytoplasm.

However, mitophagy has a dual host-protective function. Efficient removal of damaged mitochondria limits mtROS, cytosolic mitochondrial DNA, NLRP3 activation, and necrotic or pyroptotic injury. In macrophages and epithelial cells, deficient mitochondrial quality control can transform an initially antiviral response into persistent inflammation. Consequently, inhibiting mitophagy might enhance early interferon yet worsen later tissue injury. Conversely, stimulating mitophagy could suppress viral sensing early but resolve inflammation later. Therapeutic timing and cellular targeting are therefore inseparable from mechanism.

Evidence from infectious bronchitis virus adds another layer. Its spike protein inhibits FUNDC1-mediated mitophagy to prevent degradation of viral N protein [57]. Here, mitophagy is antiviral because it carries viral cargo, and the virus blocks rather than activates the pathway. Together with PDCoV and PEDV studies, these observations suggest that “mitophagy” can refer to at least two different events: removal of MAVS-bearing mitochondria, which may suppress immunity, and receptor-mediated delivery of associated viral proteins, which may restrict infection. The cargo, receptor, and stage determine the sign of the effect. A summary of selective autophagy and mitophagy mechanisms identified across different coronaviruses is presented in Table 3.

## 5. Convergence on Interferon, NF-κB, Inflammasomes, and Cell Fate

The functional importance of ubiquitin-autophagy crosstalk lies in how it redistributes innate immune output. *Coronaviruses* do not need to abolish all signaling. They need to reduce the antiviral components that impose immediate fitness costs while tolerating, or even exploiting, inflammatory pathways that damage tissue or alter host behavior.

### 5.1. Delayed Interferon Through Distributed Control

Type I and III interferon induction is attacked at multiple points. N protein can limit RIG-I activation and RNA access; PLpro removes ubiquitin or *ISG15* from sensing and signaling proteins; ORF9b inhibits TOM70; mitophagy reduces MAVS-bearing mitochondrial mass; viral proteases cleave immune and autophagy regulators; and replication organelles conceal RNA. Downstream, coronavirus proteins interfere with TBK1, IRF3, nuclear transport, and JAK-STAT signaling [2,3,4,31]. Redundancy is expected because any single antagonist may be incomplete, cell-type restricted, or opposed by high pathogen-associated molecular pattern abundance.

The distributed architecture also creates robustness against host variation. Airway epithelial cells rely strongly on MDA5 for SARS-CoV-2 sensing, whereas other cells use RIG-I as a sensor or antiviral effector. Plasmacytoid dendritic cells can detect infected cells through Toll-like receptors without productive infection. A viral strategy focused only on TRIM25-RIG-I would fail in MDA5-dominant cells; PLpro, membrane shielding, mitochondrial remodeling, and downstream blockade provide broader coverage.

The cost of this strategy becomes evident later. High viral burden and cell damage generate signals that are difficult to contain. Interferon arriving after extensive replication can recruit and activate immune cells without efficiently protecting already infected tissue. This sequence helps explain why timing, more than absolute interferon concentration, correlates with outcome. Ubiquitin and autophagy manipulations are well suited to impose such a delay because they regulate thresholds, half-lives, and compartmental access rather than simply switching genes off.

### 5.2. Selective Preservation of Inflammatory Signaling

NF-κB pathways are also ubiquitin dependent, but their regulation diverges from IRF3. Viral PLpro proteins can suppress NF-κB in some settings by removing activating chains from TRAF proteins or NEMO. In other contexts, viral proteins activate NF-κB through membrane stress, ion imbalance, linear ubiquitination, or cell injury. Specifically, coronavirus nsp14 induces NF-κB activity through linear ubiquitination by engaging LUBAC-dependent signaling [25]. Whether this represents a direct viral strategy or a host response to membrane stress remains an intriguing question. Given that nsp14 is a critical viral methyltransferase and proofreading factor, it is plausible that the virus directly exploits host linear ubiquitination machinery to deliberately drive inflammatory pathology. Alternatively, the extensive ER membrane remodeling required for DMV formation may trigger intrinsic membrane stress, indirectly activating NF-κB. It is also conceivable that these two mechanisms converge in a spatiotemporally staged manner: direct viral manipulation initiates the signaling, while host membrane stress amplifies it later during peak replication. ORF3a can promote inflammatory signaling and inflammasome activation while inhibiting autophagic flux. These apparently opposing activities may be stage specific: early suppression prevents an antiviral state, whereas later activation contributes to cytokine production and pathology.

The dual role of mitophagy in limiting inflammasome activation or suppressing MAVS-dependent interferon signaling has been discussed in Section 4.4. Whether partial mitochondrial sequestration combined with impaired lysosomal degradation can simultaneously reduce antiviral signaling and sustain inflammatory damage remains a testable model linking ORF3a-mediated flux blockade with mitochondrial immune rewiring, but direct evidence during coronavirus infection is still limited.

### 5.3. Cell Death and Tissue-Level Consequences

Ubiquitination determines the stability of apoptosis, necroptosis, and inflammasome regulators; selective autophagy determines whether damaged organelles and protein aggregates are cleared before death thresholds are crossed. Coronavirus proteins perturb both systems. Infected epithelial cells may undergo apoptosis, pyroptosis-like inflammatory death, or other lytic programs, releasing viral material and damage-associated molecular patterns. Macrophages exposed to viral products or infected-cell debris can then amplify inflammation even when viral replication is abortive.

This tissue-level division of labor is important. A mechanism that appears antiviral in an epithelial monoculture may be pathogenic in a macrophage-rich tissue, and vice versa. Enhancing lysosomal clearance in epithelium could reduce viral cargo, whereas the same intervention in antigen-presenting cells could change cytokine production and antigen presentation. Severe disease cannot be explained by a single cell-autonomous pathway. Spatial models, organoids with immune components, and in vivo lineage-specific perturbations are needed to connect molecular ubiquitin-autophagy events to physiology.

## 6. A Unifying Model: Spatially Gated and Temporally Staged Rewiring

The evidence supports a four-stage model. During entry and initial translation, the virus must avoid immediate detection. Replication RNA is shielded, N protein modifies RNA-protein condensates, and PLpro begins editing ubiquitin and *ISG15* as nsp3 is produced. At this stage, even a modest delay in interferon provides a large fitness advantage because exponential replication has not yet triggered extensive damage.

During replication-organelle expansion, nsp6 and ER quality-control machinery redirect membrane and lipid resources toward DMVs [46,47,48]. Autophagy modules may supply membranes or metabolites, but organelle size and cargo capture are constrained. Host ubiquitin systems are repurposed to regulate viral and host proteins. The virus does not need to suppress all autophagy. Instead, it must prevent productive capture of replication machinery while retaining useful membrane dynamics.

During peak replication, accumulated viral proteins intensify immune antagonism. ORF9b suppresses mitochondrial signaling, PLpro removes antiviral conjugates, and selective mitophagy or MAVS degradation reduces interferon. ORF3a blocks fusion and lysosome function, protecting captured cargo. Viral proteases may cleave autophagy receptors or initiation factors. This stage represents maximal uncoupling. Autophagosome-like structures and ubiquitinated proteins may accumulate. Yet antiviral disposal and signal propagation remain ineffective.

During late infection and tissue injury, viral control becomes incomplete. Damaged mitochondria, lysosomes, and membranes release inflammatory signals, and NF-κB and inflammasome activity may dominate over effective interferon. This stage highlights the temporal shift from early immune evasion to later inflammatory pathology, and the experimental endpoint may capture a different stage of the same trajectory. This helps reconcile apparently contradictory findings on autophagy and inflammation.

Spatial gating is equally important. Nsp3/PLpro is associated with replication membranes, ORF9b concentrates at mitochondria, ORF3a occupies endolysosomal compartments, and nsp6 resides in ER-derived membranes. Each viral protein edits a local subnetwork. Bulk proteomics or whole-cell ubiquitinomics averages these compartments and may miss decisive local changes. Proximity labeling, organelle-resolved ubiquitinomics, and live flux reporters are better suited to reveal the operative circuitry.

The model also predicts lineage-specific variation. PLpro substrate preferences differ among *coronaviruses*. Accessory proteins are poorly conserved. Animal *coronaviruses* also infect epithelial environments with distinct basal autophagy and interferon states. Conserved logic—protect replication compartments, weaken antiviral ubiquitin scaffolds, and control lysosomal disposal—can therefore be implemented by different proteins. Comparative virology is valuable for two reasons. First, not every mechanism transfers directly to SARS-CoV-2. Second, it exposes recurrent solutions and alternative molecular implementations.

## 7. Major Uncertainties and Experimental Priorities

First, the endogenous substrates of PLpro remain incompletely defined. Many proposed targets are based on candidate assays. Catalytic activity toward purified substrates does not establish access in infected cells. Quantitative diGly proteomics, *ISG15*-remnant profiling, spatial proximity methods, and recombinant viruses carrying substrate-selectivity mutations should be combined. Separation-of-function mutants that preserve viral polyprotein cleavage while altering DUB or deISGylase specificity are particularly informative.

Second, selective autophagy receptors for human coronavirus proteins are poorly mapped. Animal coronavirus studies identify NBR1, CCDC50, PGAM5-associated pathways, RBM10, and ZNF219 [50,51,52,53,54], but the receptor-cargo landscape in SARS-CoV-2 infection remains fragmentary. Genome-wide receptor screens should be performed in primary airway epithelial cells, followed by validation with endogenous proteins and infectious virus. Mapping cargo ubiquitin sites and receptor-binding determinants would reveal whether viral escape occurs through deubiquitination, proteolysis, sequestration, or membrane shielding.

Third, autophagy flux must be measured rather than inferred. LC3 puncta, LC3-II abundance, or p62 levels alone are ambiguous. Tandem fluorescent LC3 reporters, long-lived protein degradation, lysosomal pH, cargo turnover, electron microscopy, and acute genetic perturbation should be integrated. Measurements should be time resolved and performed at matched infection burden. Otherwise, reduced autophagy may simply reflect cell death or translational shutdown.

Fourth, cell identity and immune state require attention. Interferon-primed cells express *ISG15*, HERC5, immunoproteasome components, and autophagy receptors at levels unlike naive transformed cell lines. Age, metabolic disease, hypoxia, and nutrient status alter mitophagy and lysosomal capacity. These variables may explain why the same virus produces mild disease in one host and dysregulated inflammation in another. Primary cells, air-liquid-interface cultures, intestinal organoids, and immune-competent tissue models should complement permissive cell lines.

Fifth, variant evolution must be incorporated. Mutations in nsp6, N, ORF3a, ORF9b expression contexts, and proteases can alter replication-organelle formation or innate antagonism. The selective pressures acting on these proteins are not identical to those acting on spike. Comparing variants at equal intracellular viral RNA and matched time points can reveal whether immune-evasion changes are direct or simply reflect replication kinetics.

Finally, causal links to disease need in vivo resolution. A viral mutation that restores interferon may reduce replication but increase inflammation; an autophagy intervention may have different effects in epithelium and myeloid cells. Conditional host genetics and recombinant viruses should be paired with measurements of infectious burden, cytokine timing, tissue injury, and recovery. Without such integration, a molecular phenotype cannot be assumed to predict clinical benefit.

## 8. Therapeutic Implications

The therapeutic strategies discussed below differ substantially in their evidence maturity and should not be viewed as equivalent. PLpro inhibition is supported by structural, biochemical, and cell-based infection studies, and PLpro inhibitors are in preclinical or early-phase development, but no PLpro-targeted antiviral has yet been approved for clinical use. By contrast, nsp5 inhibition is supported mainly by protease-cleavage studies, and its potential to restore virophagy in human *coronaviruses* remains to be directly demonstrated. Host E3 ligase-interface targeting and restoration of selective autophagy receptors remain largely preclinical concepts, supported mainly by mechanistic cell-culture studies and requiring validation with infectious virus and primary cells. Broad autophagy modulators and mitophagy-modulating strategies remain largely speculative for coronavirus therapy, because their effects are highly context dependent and clinical trials of broad autophagy modulators in COVID-19 have not demonstrated clear benefit. We therefore explicitly distinguish experimentally supported or clinically advanced strategies from those that remain hypothetical and require further validation.

### 8.1. Viral Proteases as Dual-Purpose Targets

PLpro is attractive because inhibiting it can simultaneously block viral polyprotein processing and restore ubiquitin/*ISG15*-dependent immunity. Structural studies have enabled development of noncovalent and covalent inhibitors, including compounds related to GRL-0617 and newer chemotypes [26,27,30,58]. The therapeutic ideal is a molecule with antiviral potency, selectivity over human DUBs, suitable exposure in respiratory tissues, and activity against circulating and zoonotic *coronaviruses*. Resistance is a concern because the substrate-binding region can mutate, but the requirement for viral cleavage constrains the catalytic core. Combination with a main-protease or polymerase inhibitor could raise the resistance barrier.

PLpro inhibition may have immunological effects beyond reducing replication. Restored ISGylation and ubiquitin signaling can accelerate interferon, while altered extracellular *ISG15* may modify cytokine responses. These effects could be beneficial early but harmful if treatment begins after inflammatory disease is established. Preclinical studies should therefore compare prophylactic, early therapeutic, and late therapeutic windows and should use species models in which PLpro-*ISG15* recognition approximates the human interaction.

The main protease nsp5 is another link between replication and selective autophagy. PDCoV nsp5 cleaves NBR1 and CCDC50 [53,54], and coronavirus main proteases cleave multiple innate immune proteins. If analogous receptor cleavage occurs in human *coronaviruses*, nsp5 inhibitors may restore virophagy in addition to blocking polyprotein processing. This possibility strengthens the rationale for protease inhibition but requires direct demonstration in SARS-CoV-2-infected human cells.

### 8.2. Targeting Host Ubiquitin Interfaces

Host E3 ligase interfaces exploited by viral proteins may be druggable. Molecular glues, proteolysis-targeting chimeras, or interface inhibitors could redirect degradation toward viral proteins or protect critical host adaptors. For example, understanding Cullin 5-dependent control of ORF9b could enable selective destabilization of this mitochondrial antagonist [35]. Conversely, if a viral protein recruits STUB1 to destroy an antiviral host factor, blocking only the induced interface may preserve normal ligase function [36].

The challenge is specificity. Ubiquitin enzymes regulate thousands of substrates, and broad E1, proteasome, or Cullin inhibition is likely toxic and immunologically unpredictable. Therapeutic development should prioritize virus-induced interfaces, substrate-recruiting adaptors, or allosteric sites rather than catalytic centers shared across host physiology. Short treatment duration in acute infection may expand the safety window, but epithelial and immune-cell effects must still be resolved.

Enhancing antiviral ubiquitination is another possibility. Stabilizing TRIM25/RIPLET-RIG-I interactions or preventing viral sequestration could lower the sensing threshold. Yet excessive RIG-I or STING activation can cause damaging inflammation. Local delivery to the airway and coupling to direct-acting antivirals may reduce this risk. The objective is to restore physiological timing, not maximize cytokine output. These host-directed strategies remain preclinical and require validation in infection-competent systems.

### 8.3. Restoring Selective Autophagic Flux

Autophagy-directed therapy has often been framed as global induction or inhibition. The mechanistic evidence argues for a more precise strategy. If ORF3a blocks HOPS- and Rab7-dependent fusion, restoring this checkpoint could promote viral cargo degradation and improve lysosomal homeostasis [43,44,45]. If nsp6 restricts autophagosome expansion, disrupting the nsp6-host interface might permit capture of larger cargo without broadly activating mTOR-sensitive metabolism [47]. If viral proteases destroy receptors, cleavage-resistant receptor variants or mimetics might restore selective recognition.

General autophagy activators can be useful experimentally. However, they have complex clinical effects. mTOR inhibitors suppress translation and immune-cell growth; AMPK activators change systemic metabolism; lysosomotropic compounds may impair rather than restore flux. The failure of hydroxychloroquine in COVID-19 illustrates a danger. It shows the risk of extrapolating from in vitro entry or LC3 phenotypes to clinical efficacy. A useful therapy must be matched to viral entry route, disease stage, tissue exposure, and the specific autophagy defect.

Mitophagy modulation requires particular caution. Enhancing mitochondrial clearance may reduce inflammasome activity and tissue injury but can also remove MAVS and blunt early interferon. A staged approach could be rational: direct antivirals and restoration of sensing early, followed by support of mitochondrial and lysosomal quality control during inflammatory resolution. Biomarkers of viral replication, interferon activity, mitochondrial damage, and lysosomal function would be needed to select the appropriate phase. Restoring selective autophagic flux is therefore a mechanistically motivated but still preclinical strategy.

### 8.4. Combination Therapy and Therapeutic Timing

The network architecture favors combination therapy. A polymerase or protease inhibitor reduces viral burden; a PLpro inhibitor may additionally restore conjugation-dependent immunity; and a targeted lysosomal intervention may clear viral material and damaged organelles. Lower doses could reduce toxicity while attacking distinct failure points. However, combinations should be evaluated for immunological as well as virological synergy. Restoring interferon while blocking autophagic resolution could worsen tissue damage, whereas promoting mitophagy too early could counteract an innate agonist.

Timing can be formalized as a control problem. Early infection is dominated by exponential replication and insufficient sensing, so interventions should reduce replication and restore local antiviral signaling. Peak disease contains both virus and organelle stress, requiring balanced antiviral and cytoprotective effects. Late disease may persist despite declining infectious virus, making broad immune stimulation inappropriate; restoration of lysosomal and mitochondrial homeostasis may then be more valuable. Currently, no specific PLpro-targeted antiviral has yet been approved for clinical use, with most candidates still in preclinical or early-phase development. Similarly, broad autophagy modulators have largely failed to demonstrate clinical benefit in COVID-19, although more targeted approaches remain under investigation. These realities highlight a significant translational timeline gap between mechanistic discoveries and clinical application. Clinical trials that enroll patients solely by symptom duration may miss this biology. Viral load, interferon signatures, inflammatory markers, and markers of mitochondrial or lysosomal injury could provide a mechanistic staging framework. A summary of these therapeutic strategies and their evidence maturity is provided in Table 4.

## 9. From Association to Mechanism: A Framework for Evidence Grading

The next phase of the field should replace pathway labels with falsifiable molecular descriptions. A convincing claim that a coronavirus “uses ubiquitination” should identify several features. These include the modified substrate, linkage or architecture, relevant lysine residues, writer or eraser, subcellular site, and functional consequence. A convincing claim of selective autophagy should identify the cargo, receptor, ATG8-family interaction, lysosomal delivery, and effect on infectious yield. These criteria are demanding but necessary. Ubiquitin and autophagy measurements are unusually vulnerable to indirect effects. Viral translation arrest changes short-lived immune proteins. Cell stress changes ubiquitin pools. Lysosome damage simultaneously alters LC3, p62, metabolism, and cytokine signaling.

Evidence can be graded in four tiers. The first tier is biochemical sufficiency: purified proteins establish catalysis, binding affinity, chain preference, or protease cleavage. This tier defines molecular possibility but not cellular relevance. The second is cell-biological causality: endogenous perturbation of the ligase, DUB, receptor, or cargo site changes localization, flux, or signaling in infected cells. The third is viral genetic necessity: a recombinant virus carrying a precise separation-of-function mutation loses the phenotype without a general replication defect attributable to polyprotein processing or protein instability. The fourth is in vivo genetic necessity, which applies to both host and viral genetics: the mechanism changes viral burden, interferon timing, inflammation, or disease in primary tissue or an appropriate in vivo model. Many widely cited coronavirus mechanisms currently reach the first or second tier; relatively few satisfy all four. To apply this framework systematically, we graded representative mechanisms discussed in Section 3, Section 4, Section 5, Section 6, Section 7 and Section 8 according to the highest tier currently supported by the available evidence. SARS-CoV-2 PLpro-mediated deubiquitination/deISGylation and interferon antagonism is supported by structural and biochemical data (Tier 1) and by infection and inhibitor-rescue experiments (Tier 2), but separation-of-function viral genetics and in vivo validation remain limited; it is therefore graded as Tier 2. PLpro cleavage of ULK1 is supported mainly by biochemical and cell-based evidence and is graded as Tier 1–2, because direct evidence during SARS-CoV-2 infection remains limited. ORF3a-mediated blockade of autophagosome–lysosome fusion, ORF9b–TOM70 interaction, N-protein-mediated inhibition of RIG-I/TRIM25 signaling, nsp6-mediated autophagosome remodeling, nsp8-induced mitophagy, and nsp14-induced linear ubiquitination and NF-κB activation currently reach Tier 2, as they are supported by cell-based infection or functional assays but lack comprehensive in vivo genetic validation. In contrast, PDCoV nsp5 cleavage of NBR1/CCDC50, PEDV restriction by RBM10, PGAM5, or ZNF219, and IBV spike-mediated inhibition of FUNDC1-dependent mitophagy are supported in the respective animal *coronaviruses* at Tier 2, but their direct relevance to SARS-CoV-2 remains to be established; these findings should not be extrapolated to SARS-CoV-2 without further evidence. Overall, most of the mechanisms discussed here currently reach Tier 1–2, relatively few reach Tier 3, and none fully satisfies all four tiers. This grading clarifies which mechanisms are well supported, which are plausible but incomplete, and which require recombinant-virus, primary-cell, and in vivo validation.

Several experimental designs could accelerate progress. Time-resolved, organelle-specific proximity labeling can define which ubiquitin enzymes and receptors enter the vicinity of replication organelles, mitochondria, or ORF3a-positive endolysosomes. Linkage-selective ubiquitin binders and middle-down mass spectrometry can resolve mixed and branched chains that diGly site mapping cannot distinguish. Pulse-chase reporters can separate protein synthesis from proteasomal and lysosomal turnover. Tandem-tagged cargo, rather than LC3 alone, can show whether a viral protein reaches an acidic lysosome. Finally, barcoded recombinant-virus competition experiments can quantify small fitness effects that are obscured in high-multiplicity endpoint assays.

Interpretation should also incorporate negative results. Failure of ATG5 deletion to alter viral yield does not exclude selective autophagy because alternative ATG8 conjugation, receptor scaffolding, or noncanonical lysosomal routes may remain. Conversely, rescue by bafilomycin does not prove autophagy, because lysosomal inhibition affects endosomal traffic and viral entry. Orthogonal perturbations at initiation, cargo recognition, fusion, acidification, and proteolysis are needed to locate the responsible step. Applying this framework will reduce contradictory nomenclature and reveal which mechanisms are conserved coronavirus vulnerabilities rather than cell-line-specific epiphenomena.

## 10. Conclusions and Perspective

*Coronaviruses* exploit ubiquitination and selective autophagy as a coupled regulatory system. Viral proteins edit ubiquitin/*ISG15* signals, redirect host ligases, and remodel autophagosome biogenesis, cargo selection, mitophagy, fusion, and lysosomal degradation. The result is not simple pathway shutdown. It is selective preservation of membrane and metabolic functions that support replication, combined with weakened interferon signaling and impaired clearance of viral material.

This view resolves much of the apparent contradiction in the field. Autophagy can be antiviral when it completes selective lysosomal degradation, proviral when its membranes or mitochondrial-silencing functions are appropriated, and pathogenic when flux stalls and damaged organelles accumulate. Ubiquitination can activate antiviral signalosomes, eliminate viral proteins, degrade host defenses, or sustain inflammatory NF-κB activity, depending on chain type and location. Mechanistic claims must therefore specify substrate, linkage, receptor, compartment, flux, and infection stage.

The most promising therapeutic opportunities lie at points where viral dependence and immune antagonism converge: viral protease catalytic functions, virus-induced E3 ligase interfaces, selective-autophagy receptor escape, and viral blockade of organelle-specific flux. Progress will require genetic, spatial, quantitative, and physiologically relevant cell-based approaches. The goal is not to force maximal ubiquitination or autophagy, but to restore the timing and selectivity with which healthy cells detect, contain, and resolve coronavirus infection.

## Figures and Tables

**Table 1:** Functional logic of ubiquitin and ubiquitin-like signals in coronavirus innate immunity and selective autophagy.

Ubiquitin Signal	Representative Targets/Processes	Typical Functional Consequence	Relevance to Coronavirus Infection	Interpretive Caveat	Refs.
K48-linked/mixed chains	Immune adaptors; viral or host proteins	Proteasomal degradation; termination of signaling	Can remove antiviral proteins, but can also eliminate viral antagonists; aggregated K48-ubiquitinated cargo may enter p62-dependent autophagy	Chain type alone does not define outcome; substrate and compartment are decisive.	[5,6,7,8,9,10]
K63-linked chains	RIG-I, MAVS-associated signalosomes; damaged mitochondria	Nondegradative signaling scaffold; recruitment of selective-autophagy machinery	Supports antiviral RIG-I/TBK1 signaling, but also marks damaged organelles for autophagic capture	The same topology can support signaling or disposal depending on substrate and location.	[5,6,16,17,18,22,23,24]
M1-linked (linear) chains	NEMO/IKK and NF-κB signalosomes	Signalosome stabilization and NF-κB activation	Coronavirus nsp14 has been linked to LUBAC-dependent linear ubiquitination and NF-κB activation	Proinflammatory signaling may coexist with suppression of antiviral interferon.	[5,6,25]
K27-linked chains	STING and related signaling complexes	Regulation of innate signaling and STING activation	Contributes to ubiquitin-dependent regulation of the cGAS–STING pathway during organelle stress	Coronavirus-specific substrate-level evidence remains less complete than for major RIG-I nodes.	[5,6,19]
*ISG15* conjugation	Interferon-stimulated host proteins; PLpro substrates	Changes protein activity, localization, or stability; contributes to antiviral restriction	SARS-CoV-2 PLpro deISGylates host proteins and attenuates type I interferon responses	PLpro substrate preference and *ISG15* recognition are species dependent.	[26,27,28]

Note: IKK, inhibitor of NF-κB kinase; *ISG15*, interferon-stimulated gene 15; LUBAC, linear ubiquitin chain assembly complex; MAVS, mitochondrial antiviral-signaling protein; NEMO, NF-κB essential modulator; NF-κB, nuclear factor-κB; PLpro, papain-like protease; RIG-I, retinoic acid-inducible gene I; STING, stimulator of interferon genes; TBK1, TANK-binding kinase 1.

**Table 2:** Representative coronavirus proteins that rewire ubiquitination, autophagy, and innate immune signaling.

Virus/Viral Factor	Host Target or Pathway	Mechanism	Functional Outcome	Evidence Context	Refs.
SARS-CoV-2/PLpro (nsp3)	Ubiquitin/*ISG15*; innate signaling; ULK1	Deubiquitination/deISGylation; cleavage of ULK1	Weakens interferon signaling and can impair canonical autophagy initiation	Structural, biochemical, cell-based infection/inhibitor studies; endogenous substrate hierarchy remains incomplete	[26,27,28,29,30,31,32,33,34]
SARS-CoV-2/ORF3a	VPS39–HOPS; TBC1D5–Rab7; PTEN/STUB1	Blocks autophagosome–lysosome fusion and perturbs lysosome function; promotes STUB1-mediated PTEN degradation	Stalled autophagic flux; altered lysosomal homeostasis; suppression of antiviral signaling	Cell-based functional and infection studies	[36,43,44,45]
SARS-CoV-2/ORF9b	TOM70; Cullin 5-dependent turnover	Mitochondrial localization and TOM70 binding; host ubiquitination controls ORF9b abundance	Suppresses interferon induction; links mitochondrial antagonism to host ubiquitin control	Structural and cell-based functional evidence	[35,39,40]
SARS-CoV-2/N	TRIM25/RIG-I pathway; condensates	Interferes with RIG-I pathway activation and changes local accessibility/material state of signaling components	Reduced interferon production	Mechanistic evidence from SARS-CoV and SARS-CoV-2 systems	[37,38]
Coronavirus/nsp6	ER/autophagosome membrane machinery; ERAD	Restricts autophagosome expansion and mobilizes membrane/lipid resources	Supports replication-organelle biogenesis while limiting efficient cargo sequestration	Cross-coronavirus and SARS-CoV-2 studies	[46,47,48]
SARS-CoV-2/nsp8	Mitochondria/mitophagy	Induces mitochondrial damage and mitophagy	May reduce MAVS-bearing mitochondrial signaling and interferon output	Cell-based functional evidence	[41]
Coronavirus/nsp14	LUBAC-dependent linear ubiquitination	Promotes M1-linked ubiquitination and NF-κB activation	Preserves or enhances inflammatory signaling despite antiviral immune evasion	Cell-based functional evidence	[25]

Note: ER, endoplasmic reticulum; ERAD, ER-associated degradation; HOPS, homotypic fusion and protein sorting complex; *ISG15*, interferon-stimulated gene 15; LUBAC, linear ubiquitin chain assembly complex; MAVS, mitochondrial antiviral-signaling protein; NF-κB, nuclear factor-κB; PLpro, papain-like protease; PTEN, phosphatase and tensin homolog; RIG-I, retinoic acid-inducible gene I; STUB1, STIP1 homology and U-box containing protein 1; ULK1, unc-51-like autophagy activating kinase 1.

**Table 3:** Selective-autophagy and mitophagy mechanisms identified across representative coronaviruses.

Coronavirus	Host Factor/Receptor	Viral Cargo or Target	Mechanistic Effect	Net Consequence	Translational Relevance to SARS-CoV-2	Refs.
PEDV	RBM10	nsp3	Promotes selective autophagic degradation of nsp3	Antiviral	Comparative evidence; corresponding role in SARS-CoV-2 human cells remains unestablished	[50]
PEDV	PGAM5-associated pathway	N protein	Promotes selective autophagy-mediated degradation of N	Antiviral	Comparative evidence only	[51]
PEDV	ZNF219	S2 protein	Promotes degradation of viral S2 protein	Antiviral	Comparative evidence only	[52]
PDCoV	NBR1	E protein	Recognizes E and directs it toward autophagic degradation; nsp5 cleaves NBR1	Host antiviral defense counteracted by viral protease	Strong mechanistic precedent for receptor-directed virophagy, but not established for SARS-CoV-2	[53]
PDCoV	CCDC50	E protein	Targets E for autophagic degradation; nsp5 cleaves CCDC50	Host antiviral defense counteracted by viral protease	Comparative evidence; highlights receptor escape as a potential conserved strategy	[54]
PDCoV	Mitophagy machinery	Mitochondria/MAVS-associated signaling	Infection triggers mitophagy	Dampens interferon and promotes replication	Supports dual-role model of mitophagy; cross-genus extrapolation requires caution	[42]
IBV	FUNDC1-mediated mitophagy	N protein associated with mitochondrial quality-control pathway	Spike inhibits FUNDC1-mediated mitophagy and prevents N degradation	Proviral blockade of an antiviral mitophagy route	Demonstrates that mitophagy can be antiviral when viral cargo is coupled to mitochondrial clearance	[57]
SARS-CoV-2	Autophagy/mitophagy machinery	MAVS-bearing mitochondria	Promotes autophagy-dependent MAVS degradation and nsp8-associated mitophagy	Suppresses type I interferon	Human-coronavirus evidence, but therapeutic direction is stage dependent	[41,56]

Note: IBV, infectious bronchitis virus; MAVS, mitochondrial antiviral-signaling protein; PDCoV, porcine deltacoronavirus; PEDV, porcine epidemic diarrhea virus; SARS-CoV-2, severe acute respiratory syndrome coronavirus 2. Findings from animal coronaviruses are comparative evidence and should not be directly extrapolated to SARS-CoV-2 without additional validation.

**Table 4:** Therapeutic opportunities arising from coronavirus rewiring of ubiquitin and selective autophagy.

Therapeutic Strategy	Molecular Rationale	Expected Benefit	Evidence Maturity	Major Limitation/Risk	Refs.
PLpro inhibition	PLpro couples viral polyprotein processing with deubiquitinating and deISGylating activities.	Suppress viral replication while restoring ubiquitin/*ISG15*-dependent early innate signaling.	Relatively advanced preclinical evidence; structural, biochemical, infection and inhibitor-rescue studies; no approved PLpro-targeted antiviral.	Selectivity over host DUBs; resistance; species-dependent PLpro–*ISG15* recognition; late interferon restoration could worsen inflammation.	[26,27,30,58]
nsp5 inhibition to preserve selective-autophagy receptors	PDCoV nsp5 cleaves NBR1 and CCDC50, disrupting selective autophagic degradation of viral E protein.	Direct protease inhibition plus potential restoration of virophagy.	Mechanistically supported in animal coronaviruses; human-coronavirus virophagy restoration remains unproven.	Cannot yet be directly extrapolated to SARS-CoV-2; relevant receptor substrates in human infection remain incompletely defined.	[53,54]
Target virus-induced host E3 ligase–substrate interfaces	Coronavirus proteins redirect host ligases; examples include Cullin 5 control of ORF9b and ORF3a-associated STUB1-mediated PTEN degradation.	Destabilize viral antagonists or protect antiviral host factors while preserving normal ubiquitin-system function.	Preclinical/mechanistic.	Host E3 ligases have many physiological substrates; virus-specific interfaces must be selectively targeted.	[35,36]
Enhance antiviral ubiquitination	Stabilize productive TRIM25/RIPLET–RIG-I interactions or prevent viral disruption of activating ubiquitin scaffolds.	Lower viral-RNA sensing threshold and accelerate interferon induction.	Conceptual/preclinical.	Excessive RIG-I/STING activation may amplify damaging inflammation; tissue- and stage-specific delivery may be needed.	[16,17,18]
Restore autophagosome–lysosome fusion	ORF3a disrupts VPS39/HOPS-dependent fusion and TBC1D5–Rab7-regulated lysosomal function.	Restore flux, viral-cargo degradation, and lysosomal homeostasis.	Mechanistically supported; therapeutic implementation remains preclinical.	Manipulating lysosomal trafficking can affect many cellular processes; efficacy is stage and cell-type dependent.	[43,44,45]
Counteract nsp6-mediated autophagosome remodeling	nsp6 restricts autophagosome expansion and can engage ERAD machinery to mobilize lipids for DMV formation.	Improve cargo sequestration while limiting membrane/lipid support for replication organelles.	Preclinical/mechanistic.	Relevant host interfaces remain incompletely defined; membrane/autophagy pathways are essential to normal homeostasis.	[46,47,48]
Restore selective-autophagy receptor function	NBR1, CCDC50, RBM10, PGAM5-associated pathways, and ZNF219 mediate selective degradation of viral proteins in animal coronaviruses.	Selective elimination of viral proteins with less disruption of bulk autophagy.	Early preclinical/conceptual; strongest evidence from PEDV, PDCoV and IBV.	Corresponding receptor–cargo relationships in SARS-CoV-2-infected human cells remain largely undefined.	[50,51,52,53,54,57]
Mitophagy modulation	Mitophagy can remove MAVS-bearing mitochondria and suppress interferon, but can also limit mtROS, mtDNA release and NLRP3-driven injury.	Stage-dependent preservation of antiviral sensing early and support of inflammatory resolution later.	Context-dependent and largely speculative therapeutically.	Potentially opposite effects at different stages; activation may suppress early interferon whereas inhibition may aggravate late inflammatory injury.	[41,42,56,57]
Broad autophagy modulation	mTOR, AMPK, VPS34, lysosomal and related pathways alter autophagy and coronavirus replication.	Potential modification of replication, membrane metabolism or cellular clearance.	Low translational confidence for broad modulation.	Extensive pleiotropy; broad approaches have not shown clear clinical benefit in COVID-19.	[55]
Mechanism-guided combination therapy	Combine direct antivirals with restoration of conjugation-dependent immunity and targeted lysosomal/autophagy interventions.	Attack replication, immune evasion and defective clearance at distinct nodes; potentially lower individual drug doses.	Conceptual/preclinical framework.	Poorly timed components may be antagonistic; biomarker-guided staging would be required.	[43]

Note: AMPK, AMP-activated protein kinase; DMV, double-membrane vesicle; DUB, deubiquitinating enzyme; ERAD, ER-associated degradation; HOPS, homotypic fusion and protein sorting complex; *ISG15*, interferon-stimulated gene 15; MAVS, mitochondrial antiviral-signaling protein; mtDNA, mitochondrial DNA; mtROS, mitochondrial reactive oxygen species; NLRP3, NLR family pyrin domain containing 3; PLpro, papain-like protease; RIG-I, retinoic acid-inducible gene I; STING, stimulator of interferon genes; VPS34, vacuolar protein sorting 34. Evidence maturity differs substantially among strategies; broad autophagy and mitophagy modulation remain highly context- and stage-dependent.

## Data Availability

No new data were created or analyzed in this study. Data sharing is not applicable to this article.
